# Genetics in human reproduction

**DOI:** 10.5935/1518-0557.20200007

**Published:** 2020

**Authors:** Vivian de Oliveira Rodrigues, Fernanda Polisseni, Gabriel Duque Pannain, Miralva Aurora Galvão Carvalho

**Affiliations:** 1 Federal University of Juiz de Fora, Juiz de Fora, MG, Brazil; 2 Surgery Department, Medical School - Federal University of Juiz de Fora, Juiz de Fora, MG, Brazil

**Keywords:** genetics, male infertility, female infertility, preimplantation genetic diagnosis, preimplantation genetic screening

## Abstract

Approximately 50% of the causes of infertility are of genetic origin. The objective of this study was to analyze the role of genetics in human reproduction by reviewing the main genetic causes of infertility and the use of preimplantation genetic testing in Brazil. This literature review comprised articles in English and Portuguese published on databases PubMed, Scielo, and Bireme from 1990 to 2019. Randomized clinical trials and specialized guidelines were given preference whenever possible. Genetic cause can be traced back to up to 20% of the cases of severe azoospermia or oligozoospermia. Subjects with these conditions are good candidates for genetic screening. In women, genetic causes of infertility (fragile X syndrome, X-trisomy, and Turner's syndrome, some of which diagnosed with karyotyping) culminate with premature ovarian failure. Genetic screening helps advise couples of the risk of experiencing early reproductive capacity loss and of the chances of their offspring carrying genetic disorders. In addition to enhancing the prevention of serious diseases in the offspring of couples at increased risk of genetic diseases, preimplantation genetic screening improves the success rates of assisted reproduction procedures by allowing the selection of euploid embryos for transfer. The interface between genetics and human reproduction has gained significant relevance, but discussions are still needed on which procedures are clinically and ethically acceptable and how they should be regulated.

## INTRODUCTION

According to the World Health Organization, infertility is a disease of the reproductive system defined by the inability of sexually active couples to get pregnant within a period of one year, without the use of contraceptive methods. In women over 35 years of age or couples with known infertility-related comorbidities, this period is six months (Marshburn, 2015; Zegers-Hochschild *et al*., 2009).

Genetic and environmental factors may be related to infertility, which often has a multifactorial etiology. It has been estimated that approximately 50% of the causes of infertility are related to genetic factors. Hundreds of experimental studies with animal models have demonstrated an association between infertility and single or multiple gene defects. Despite these advances, translating these results into clinical trials has been challenging. At present, only a small number of genes and genetic changes have been unequivocally associated with primary infertility. This situation has been changing since the conclusion of the genome project and the progress of personalized medicine. In fact, ten to 15 new genetic tests are added to the roster of genetic tests annually (Zorrilla & Yatsenko, 2013).

The main known genetic causes of infertility include chromosomal aberrations, monogenic diseases, and phenotypes with multifactorial inheritance. The physiology of reproduction involves several paracrine, autocrine, and endocrine processes. They are regulated by a plethora of genes and any discrepancy in these processes can lead to infertility (Venkatesh *et al*., 2014).

In men, fertility criteria include normal spermatogenesis, complete sperm maturation during passage through accessory organs of the reproductive system, patency of accessory organs, adequate production of seminal fluid, ability to deposit semen into the vagina, adequate sperm cell mobility and morphology so it can reach the oocyte in the uterine tubes and penetrate it (Travaglini *et al*., 2006).

The main genetic causes of male infertility are chromosomal abnormalities, mutation in the cystic fibrosis transmembrane receptor (CFTR) gene, and microdeletion on the Y chromosome. Genetic cause can be traced back to up to 20% of the cases of severe azoospermia or oligozoospermia. Subjects with these conditions are good candidates for genetic screening. Although the detection of genetic alterations does not substantially alter treatment, they must be analyzed for two main reasons: to achieve a conclusive causal diagnosis and assess the genetic risk to the offspring in case of successful treatment (Kara & Simoni, 2010).

In women, genetic causes of infertility (fragile X syndrome, X-trisomy, and Turner's syndrome, some of which diagnosed with karyotyping) culminate with premature ovarian failure. Complex multifactorial conditions such as endometriosis and polycystic ovary syndrome have been associated with gene alterations (Zorrilla & Yatsenko, 2013).

Recurrent miscarriage, defined as three or more pregnancy losses before 20 weeks of gestation, may also have a genetic etiology. Between a quarter and 51% of the cases of recurrent miscarriage have been associated with chromosomal anomalies of the fetus. Karyotyping of miscarriage products should be performed to determine the cytogenetic reasons for the pregnancy loss. Karyotyping of the parents is also recommended (Kara & Simoni, 2010).

Genetic testing has applications not only in the investigation of infertile couples, but also in preimplantation analysis before in vitro fertilization (IVF). Preimplantation genetic testing (PGT) is a clinical application of genetics that enables the examination of a limited number of embryonic cells during their *in vitro* development (Harper *et al*., 2018; Zegers-Hochschild *et al*., 2009).

Many recent studies in the field of genetics have mentioned the transition from traditional "monogenic genetics" to comprehensive testing of the human genome through the integration of Next Generation Sequencing (NGS), which allows complete DNA sequencing in a single day with bioinformatics techniques. Novel technologies have shed light on the variations of the human genome and extended the application of genetics. Recent technological advances are already being used to investigate the underlying causes of male and female infertility and in preimplantation genetic testing, both subjects of this paper (Harper *et al*., 2018).

## MATERIAL AND METHODS

This literature review comprised articles in English and Portuguese published on databases PubMed, Scielo, and Bireme from 1990 to 2019. Additional references were collected from relevant studies. Randomized clinical trials and specialized guidelines were given preference whenever possible. The search yielded 122 papers from randomized controlled trials, guidelines of renowned medical societies in the field, or review articles, all of which read in full. The search used keywords genetics, male infertility, female infertility, preimplantation genetic test, preimplantation genetic diagnosis, and preimplantation genetic screening.

## RESULTS

### Male infertility and genetics

Keywords genetics and male infertility found 11,985 matches. Only papers whose full version was available and recent publications from renowned authors on relevant themes were read. Table 1 lists the main monogenic diseases associated with male infertility (Asero *et al*., 2014).

**Table 1:** Main genes associated with male infertility

Gene	Disease	Clinical aspects
CFTR	congenital bilateral absence of the vas deferens	Obstructive azoospermia
KAL-1	Kallmann syndrome	Hypogonadotropic hypogonadism and changes in spermatogenesis
AR	Androgen insensitivity syndrome	Decreased androgen sensitivity and changes in spermatogenesis
INSL3-RXFP2	Cryptorchidism	Changes in spermatogenesis

Source: Asero *et al*., 2014.

### Klinefelter syndrome

Klinefelter syndrome (KS) is the most common cause of male infertility of genetic origin. Its prevalence reaches 5% in men with severe oligozoospermia and increases up to 10% in subjects with spermogram-documented azoospermia. Infertility occurs due to changes in spermatogenesis and testicular injuries in individuals with progressive syndrome, progressive fibrosis, degeneration of germinal cells and Sertoli cells (Bonomi *et al*., 2017; Klinefelter *et al*., 1942; Lanfranco *et al*., 2004; Piomboni *et al*., 2014; Wosnitzer & Paduch, 2013).

There is consensus around the correlation between testicular phenotype severity and frequency of chromosomal abnormalities such as KS. For this reason, G-banding is recommended in Europe for men with sperm concentrations of less than 10 million/mL. This cut-off point was established based on the fact that the incidence of chromosomal alterations is ten times higher in these patients compared with the general population (Jungwirth *et al*., 2012; Krausz & Chianese, 2014).

The American Society for Reproductive Medicine recommended karyotyping to screen males with non-obstructive azoospermia (absence of azoospermia) or severe oligozoospermia (concentration <5 million/mL) for KS, especially before ICSI (ASRM, 2015).

Peripheral blood karyotyping and identification of KS are fundamental in the differential diagnosis of obstructive azoospermia (sperm production is normal, but there is no sperm in the ejaculate due to obstruction or absence of the vas deferens) and non-obstructive azoospermia (no or minimal sperm production as in KS). In this case, genetic testing is a valuable tool to achieve proper patient management (Wosnitzer *et al*., 2014).

The treatment of this syndrome requires a multidisciplinary approach and the involvement of assisted reproduction specialists. A small proportion of men with KS maintain capacity for spermatogenesis at levels to allow the presence of spermatozoa in ejaculate. However, men with KS may have residual preserved spermatogenesis and testicular sperm extraction (TESE) may retrieve tubules with active spermatogenesis. The combination of TESE and ICSI, in which the spermatozoon is injected directly into the egg, allowed men with KS previously considered sterile to father children (Vloeberghs *et al*., 2018).

### Robertsonian and reciprocal translocations

Robertsonian translocations are structural chromosomal abnormalities that cause infertility by altering the genetic pattern of spermatozoa. They result from the fusion of the long arms of two acrocentric chromosomes (13, 14, 15, 21, 22) to form an anomalous chromosome. A reciprocal translocation, on the other hand, occurs when genetic material is exchanged usually between non-homologous chromosomes (Asero *et al*., 2014).

Balanced reciprocal translocations do not cause changes in carrier phenotype. However, in some cases they may cause decreases in testicular volume and testosterone levels, impacting spermatogenesis and resulting in azoospermia or oligozoospermia (Godo *et al*., 2013).

Individuals with severe oligozoospermia and azoospermia and couples experiencing recurrent miscarriages should undergo karyotyping to find possible chromosomal alterations. Furthermore, when a spermatozoon carrying a chromosome translocation fertilizes an ovum, the resulting embryo will carry the translocation, generating a genetic imbalance. The associated phenotype will depend on the exact region of the chromosome involved, which may result in mental retardation, malformations, and death of the fetus. When a translocation is detected in one of the spouses, the couple may choose to order preimplantation genetic screening of the embryos, embryo biopsy, or PGT to rule out the presence of the translocation in the embryos (Asero *et al*., 2014; Yin *et al*., 2017).

### Y chromosome microdeletions

Azoospermia factor (AZF) was identified in the long arm of the Y chromosome in 1996 and deletions in this region were identified in 13 of 370 men with severe oligozoospermia or azoospermia. Although microdeletions are too small to be found in conventional karyotyping, they can be diagnosed via polymerase chain reaction (ASRM, 2015; Vogt *et al*., 1996).

AZF regions are divided into AZFa (proximal), AZFb (central), and AZFc (distal) and contain many of the genes needed in spermatogenesis. Males with deletions in the AZFc region may have sperm in ejaculate. Some with the same deletion are azoospermic, but may have sufficient sperm production to allow TESE. TESE is contraindicated for patients with deletions in the AZFa and AZFb regions, since their chances of having a successful sperm extraction procedure are extremely slim (Hopps *et al*., 2003; Oates *et al*., 2002).

The frequency of Y chromosome microdeletions ranges from 1% to 58% in published studies, and more specifically from 15% to 20% in males with idiopathic non-obstructive azoospermia; 7-10% in males with idiopathic oligozoospermia (sperm counts of less than 5 million/mL); and 2-3% in ICSI candidates. Differences in frequency might be due to poor patient selection, differences in the ethnicity of the studied population, sample size, and differences in study design (Li *et al*., 2008; Rives, 2014; Suganthi *et al*., 2014).

Thus, analysis of Y chromosome microdeletions based on peripheral blood should be offered to men with non-obstructive azoospermia or severe oligozoospermia before ICSI. In cases of non-obstructive azoospermia, the test may not only identify the origin of spermatogenesis impairment, but also predict the probability of sperm retrieval after TESE (ASRM, 2015; Rives, 2014).

In addition, considering that sperm counts may be significantly reduced with aging in men with Y chromosome microdeletions, sperm cryopreservation should be offered at the time of diagnosis when sperm cells are present in the ejaculate (Rives, 2014).

Consequently, when ICSI is performed in patients with Y chromosome microdeletions, the couple should be advised of the risk of transmitting the condition to their male offspring along with its negative effects on spermatogenesis. Therefore, couples should be instructed to order semen analysis (spermogram) for their adolescent children to consider the possibility of cryopreserving sperm as a measure of fertility preservation (ASRM & SMRU, 2018; Rives, 2014).

### Cystic fibrosis and other monogenic diseases

The main known monogenic disease is congenital bilateral absence of the vas deferens (CBAVD) with obstructive azoospermia. Mutations in the CFTR gene are found in more than 90% of the patients with agenesis of the vas deferens (Zorrilla & Yatsenko, 2013).

The CFTR gene is located on chromosome 7. In its homozygous form, the gene causes cystic fibrosis, one of the most common and severe autosomal recessive diseases to affect Caucasians. One in 2,500 individuals is affected and one in 25 is an asymptomatic carrier of mutation. The presence of mutations that do not completely impair the expression of the CFTR gene causes CBAVD in men, with consequent obstructive azoospermia. CBAVD is found in 6% of the patients with obstructive azoospermia and in about 2% of infertile individuals. Infertility caused by obstructive azoospermia is observed in more than 95% of the males with cystic fibrosis, while 60-70% of the patients with CBAVD have mutations on the CFTR gene without manifesting clinical symptoms of cystic fibrosis (Asero *et al*., 2014; Claustres *et al*., 2000).

Individuals with obstructive azoospermia are candidates for mutation testing on the CFTR gene through peripheral blood analysis, since they may present a congenital malformation of the Wolff ducts, which are precursors of the vas deferens, epididymis, and seminal vesicles during fetal development (Tüttelmann & Simoni, 2008). Most of these patients have normal spermatogenesis observed in testicular biopsy. Thus, there is a significant chance that these men might have children through ICSI. However, since the offspring of these couples is at risk of cystic fibrosis when the female partner is heterozygous for the CFTR gene, screening for this mutation is imperative in humans before attempting assisted reproduction technology procedures (Field & Martin, 2011; Tüttelmann & Simoni, 2008).

### Female infertility and genetics

The search for papers under this item used keywords genetics and female infertility and yielded 8805 matches. However, the authors read only papers available in full and prioritized recent publications with relevant themes and authors.

### Premature ovarian failure (POF)

The end of a woman's reproductive life is marked by the occurrence of menopause, defined as the last menstruation, caused by the exhaustion of the ovarian reserve. In the general female population, menopause occurs at 50-52 years of age. However, changes in ovulation may cause a pathogenic depletion of the ovarian follicles, resulting in early menopause. Menopause before the age 40 is the definition of POF (Perry *et al*., 2013; Shelling, 2010).

POF has been described as the premature cessation of ovarian function. The condition is characterized by 4-6 months of amenorrhea, increased levels of FSH (above 40,000/L), and hypoestrogenism. It occurs in 1% of all women and in 0.1% of women under the age of 30. POF has been associated with increased risk of osteoporosis, osteoarthritis, and cardiovascular disease, all of which related to hypoestrogenism. In addition to experiencing typical postmenopausal symptoms, women with POF suffer from early loss of reproductive capacity. Therefore, women at high risk for POF and who delay pregnancy to after the age of 30 may experience difficulty conceiving and maintaining pregnancy to term (Chapman *et al*., 2015; Perry *et al*., 2013).

A 2011 study showed that 50-90% of the causes of POF are idiopathic and probably have a significant genetic contribution. A genetic etiology of premature ovarian failure has been reinforced by estimates that between 44-65% of the daughters of mothers with FOP also have the condition. Recent reports have described various age-related genetic loci in natural menopause identified through broad genomic association studies. It is also important to note that in 10-30% of idiopathic cases a first-degree relative is affected. In addition, daughters of mothers with POF have a six-fold risk of manifesting the disease. The genetic causes of POF include chromosomal abnormalities, gene mutations (Table 2), and gene polymorphisms (Cordts *et al*., 2011; He *et al*., 2009; Pu *et al*., 2014; Qin *et al*., 2012; Stolk *et al*., 2009). However, the genetic alterations identified to date account a small proportion of the cases of POF. The disease has a diverse and heterogeneous etiology, involving the interaction of multiple genes, environmental factors, and associations with autoimmune conditions (Dixit *et al*., 2010; Qin *et al*., 2012).

**Table 2:** POF-associated genes

Genes involved in ovarian function	Genes involved in oogenesis
FSH / FSHR	NOBOX LH / LHR LHX8
CYP17	NANOS CYP19
BMP15	
GDF9	
GPR3	

Source: Kara & Simoni, 2010.

Given the multifactorial etiology of POF, patients suspected for the disease should not undergo genetic testing. The associations described between the condition and a few noteworthy genetic diseases - fragile X syndrome (mutation in the FMR-1 gene on X chromosome at Xq27), chromosome X trisomy (47, XXX), and Turner’s syndrome (monosomy of chromosome X; 45,X0), to name a few - are still controversial. (Abir *et al*., 2001; Barasoain *et al*., 2013; CFM, 2017; De Geyter *et al*., 2014; Gleicher *et al*., 2010; Kawamura *et al*., 2016; Lubs, 1969; Schufreider *et al*., 2015; Sullivan *et al*., 2005; Tartaglia *et al*., 2010).

### Other Causes

Other important causes of female infertility also appear to be linked to genetic alterations, including the likes of polycystic ovarian syndrome (PCOS) and endometriosis.

Although PCOS appears to follow a pattern of dominant inheritance, no specific gene has been linked to the disease. Therefore, genetic testing for this condition loses its purpose. There is a known association between PCOS and the following genes: FBN3, FST, INS, INSR, TCF7L2, CAPN10, FTO, SHBG, PCOS1, SRD5A1, SRD5A2, and CYP11A. However, they have also been associated with obesity, diabetes, and insulin resistance, alterations commonly associated with PCOS. To date, only the insulin receptor gene (INSR) has demonstrated a more significant association with susceptibility to PCOS as described in the GWAS study (Chen *et al*., 2011; Kosova & Urbanek, 2013).

The presence of disease in first-degree relatives increases the risk of endometriosis by five to eight times. In 1999, a study described gene changes in three chromosomal regions (1p36,7p22.1 and 22q1) in patients with endometriosis (Bulun, 2009; Gogusev *et al*., 1999; Painter *et al*., 2011; Treloar *et al*., 2005; Uno *et al*., 2010). It has been recently suggested that retinoid deficiency plays a causal role in the etiology of endometriosis. Abnormal methylation of the promoters of genes such as GATA6, ESR2 and NR5A1 in endometrial implants leads to local increases in estrogen and prostaglandin levels, causing inhibition of progesterone receptors. This, in turn, results in the reduction of retinoid synthesis and absorption. These molecular abnormalities have detrimental effects on cell differentiation and produce excessive inflammation, which might result in the development of endometriosis (Bulun *et al*., 2015).

### Embryo biopsy and preimplantation genetic testing

A search using keywords preimplantation genetic diagnosis and preimplantation genetic screening yielded 221 matches. However, we read only the papers available in full text format. Priority was given to more recent texts of prominent authors discussing relevant themes.

Genetic analysis is not valuable only in the investigation of infertile couples; it also allows the analysis of embryo diseases before implantation via assisted reproduction procedures. Preimplantation genetic testing (PGT) is a clinical application of genetics that allows the examination of a limited number of embryo cells harvested by biopsy during in vitro embryo development. Assisted reproduction procedures involve the in vitro management of oocytes, spermatozoa or human embryos with the objective of achieving pregnancy (Zegers-Hochschild *et al*., 2009).

There currently are two types of PGT: preimplantation genetic testing for monogenic diseases (PGT-M) and preimplantation genetic testing of aneuploidies (PGT-A). PGT-M is designed to diagnose a specific Mendelian genetic disorder in the embryo for which the parents are at high risk, as in the case of multiple sclerosis. PGT-A is used to detect chromosomal aneuploidies (chromosome number alterations) and select embryos free from conditions such as Down syndrome (trisomy 21) (Farquhar & Marjoribanks, 2018; Traeger-Synodinos, 2017).

Likewise, there have been significant advances in techniques used for genetic and chromosomal analysis using small amounts of DNA, including polymerase chain reaction (PCR), fluorescence in situ hybridization (FISH), single nucleotide polymorphism (SNP) microarrays, comparative genomic hybridization (aCGH), and new generation sequencing (NGS) (Sullivan-Pyke & Dokras, 2018).

### Embryo biopsy techniques

Polar body biopsy

The procedure involves the removal of the first and second polar bodies (cells resulting from meiosis I and II, which occur during oogenesis) prior to the initiation of embryo cleavage. Although polar body biopsy precludes the removal of embryo cells, it is limited by the fact that only maternal genes and chromosomes can be analyzed, thus excluding possible paternal contributions to the embryo. In addition, the amount of material obtained from a single cell is small and subject to limitations. For these reasons, this technique is not widely used (Sullivan-Pyke & Dokras, 2018; Verlinsky *et al*., 1990).

### Blastomere biopsy from cleavage-stage embryos

The procedure consists of the removal of one or two cells (blastomeres) from an embryo in the cleavage stage, which has between six and eight cells. This technique is more advantageous than polar body biopsy, since maternal and paternal contributions can be analyzed. However, its limitations include the small amount of genetic material available for study and the presence of mosaicism (Treff & Franasiak, 2017).

The presence of mosaicism in early-stage embryos is significant. A 1994 study described a 50% rate of mosaicism in cleavage-stage embryos. Recent studies claim that the proportion may be as high as 60%. Therefore, it is possible that the cell biopsied and tested during PGT-A might not represent the ploidy status of other embryo cells. Mosaicism potentiates the occurrence of diagnostic error and undesired clinical outcomes even in cases where a precise cellular genetic diagnosis has been performed (Brezina *et al*., 2016a; 2016b; Capalbo *et al*., 2013; Munné *et al*., 1994).

In addition to the high rates of mosaicism, removing cells at the cleavage stage may delay the development of the embryo to the blastocyst stage and decrease implantation rates and pregnancy (Scott *et al*., 2013a, 2013b).

### Trophectoderm biopsy

The first birth after trophectoderm biopsy and blastocyst-stage embryo transfer was reported in 2005, a decade after the first reports of births from blastomere biopsies (Kokkali *et al*., 2005). The development of sequential culture media allowed the success of embryo culture at the blastocyst stage (extended culture) and improved gestation rates after blastocyst transfer. The introduction of trophectoderm biopsy in clinical practice has allowed the analysis of hundreds of cells with consequent accuracy improvements, since there is excellent genetic agreement between the internal cell mass of the embryo and the trophectoderm. Although lower rates of mosaicism have been described in blastocyst-stage biopsies compared with cleavage-stage biopsies, mosaicism confined within this layer as described in the placenta in later stages, or variations within the trophectoderm itself may lead to erroneous results (Gardner *et al*., 1998).

### PGT-A

Aneuploidy is a common event in developing human embryos. It has been defined as any number of chromosomal copies other than diploidy affecting any of the 23 pairs of chromosomes. Examples include trisomy (an extra copy of a chromosome) and monosomy (one copy less). It is currently believed to occur in most embryos. Frequency of aneuploidy increases with maternal age. Aneuploidies are the most common cause of early miscarriage and usually halt embryo development before implantation (Brezina *et al*., 2012; Brezina & Kutteh, 2015; Ginsburg *et al*., 2011; Maxwell *et al*., 2016).

Pregnancy rates from IVF may be improved with the transfer of only euploid embryos to the uterus, resulting in higher implantation and lower miscarriage rates. PGT-A is an option for patients undergoing IVF and a particularly useful tool for couples with an aging female partner, individuals with a history of recurrent first trimester pregnancy loss, and subjects with recurrent implantation failure in previous cycles of IVF (Sullivan-Pyke & Dokras, 2018).

FISH was initially used with in single-cell biopsies to evaluate a limited number of chromosomes most frequently associated with aneuploidy. However, in 2007 a prospective study reported that PGT-A did not increase pregnancy rates. Other authors have since found no benefit from PGT-A in terms of improved pregnancy outcomes. Therefore, the American Society for Reproductive Medicine (ASRM), the American College of Obstetrics and Gynecology (ACOG), and the European Society for Human Reproduction and Embryology (ESHRE) issued formal opinions discouraging the use of PGT-A (ACOG, 2009).

However, the development of single-cell genome amplification allowed the use of new technologies to quantify all 24 chromosomes, known as comprehensive chromosome tracking (CCS), which includes microarrays with SNP matrices, aCGH, PCR, and NGS (Table 3) (Brezina *et al*., 2016a; 2016b;Sullivan-Pyke & Dokras, 2018). The clinical validation of the technologies involved in PGT-A must include an assessment of pregnancy and live birth rates (Sullivan-Pyke & Dokras, 2018).

**Table 3:** Comparison of chromosomal genetic tests

Method	Duration	Anomalies	Limitations
Array Comparative Genomic Hybridization (aCGH)	12 hours	Aneuploidies Translocations	False positives. It does not detect mosaics.
Single Nucleotide Polymorphism Array (SNP)	72 hours	Aneuploidies Translocations Parental Origin	Does not detect balanced translocations and mosaics.
Quantitative Polymerase Chain Reaction (qPCR)	4 hours	Aneuploidies	Does not detect segmental aneuploidies, translocations and mosaics.
Next-Gen Sequencing (NGS)	< 24 hours	Aneuploidies Mosaics Monogenic Diseases Translocations	Limited capacity to detect balanced translocations

Source: Sullivan-Pyke & Dokras, 2018.

A 2017 study showed that the transfer of euploid blastocysts identified after PGT-A by aCGH increased implantation (52.8% *vs.* 27.6%) and live birth (64.8% *vs.* 27.4%) rates compared with untested transferred blastocysts (Rubio *et al*., 2017). A retrospective study published in 2012 showed that the transfer of a single euploid embryo identified by trophectoderm biopsy with qPCR resulted in higher pregnancy (55% *vs*. 41.8%) and lower miscarriage (10.5% *vs.* 24.8%) rates compared with untested embryo transfers (Forman *et al*., 2012). In a randomized clinical trial with 72 patients submitted to qPCR biopsy, implantation rates were higher in the case (79.8%) than in the control group (63.2%) (relative risk [RR] 1.26; 95% CI 1.04-1.39, *p*=0.002), and the proportions of live births were 66.4% and 47.9%, respectively (RR 1.39, 95% CI 1.07-1.60; *p*=0.001) (Scott *et al*., 2013a, 2013b).

There are advantages and disadvantages to screening for aneuploidies in embryos. The procedure is known to reduce the risk of aneuploidies detected during pregnancy and after birth. In addition, the identification and subsequent disposal of aneuploid embryos may decrease the cost of excess frozen embryos (ASRM & SMRU, 2018). When used to identify euploid embryos, PGT-A may shorten the time to pregnancy and allow the selection of embryos with greater chances of implantation. The procedure benefits older women, couples willing to have more children, and cancer patients. In addition, it offers the possibility of selecting the sex of the embryo (ASRM & SMRU, 2018). Potential drawbacks of the method include the need for increased resources and the use of up to eight cumulative hours of work for the embryology team in each biopsy. Not every embryo survives in culture media to the blastocyst stage required for trophectoderm biopsy. However, they might have hypothetically resulted in live births if they had been transferred in the initial cleavage or blastocyst stage (Alikani *et al*., 2014; ASRM & SMRU, 2018).

Given the uncertainties around the supposed ability embryos have to autocorrect, the false-positive rates of PGT-A, and the accuracy of diagnoses of mosaicism, there is concern that embryos that might result in healthy babies are being discarded (Greco *et al*., 2015). A number of authors have looked into factors such as cost-effectiveness, time to gestation, use in specific subgroups of patients (recurrent miscarriage, previous implantation failure, advanced maternal age), and cumulative success rates tied to PGT-A. The American Society of Reproductive Medicine does not recommend the routine use of PGT- A in infertile patients (ASRM & SMRU, 2018). However, some patient subpopulations benefit from PGT-A, including couples experiencing unexplained recurrent pregnancy loss, couples with recurrent aneuploidy as the cause of miscarriage, couples with repeated implantation failures in IVF cycles, men with severe male factor infertility, couples undergoing PGT-M, and couples in fertility treatment looking for single-embryo transfers (Sullivan-Pyke & Dokras, 2018).

### PGT-M

Preimplantation genetic diagnosis determines whether embryo cells carry genetic anomalies associated with specific disorders known to affect one or both parents (Brezina & Kutteh, 2015). Gardner & Edwards (1968) were the first to publish on biopsies of trophectoderm cells of blastocyst-stage rabbit embryos to identify sex. This seminal animal study set the stage for further studies involving human embryo biopsy and PGT-M (Gardner & Edwards, 1968; Sullivan-Pyke & Dokras, 2018).

The first successful case of preimplantation genetic testing in humans was in fact a PGT-M. In 1990, the test was performed for adrenoleukodystrophy, an X-linked recessive condition. The cleavage-stage embryos were biopsied and PCR was performed to distinguish between male and female embryos. The female embryos were transferred and yielded two pregnancies. In 1992, PGT-M was performed after cleavage-stage embryo biopsy to detect a specific mutation associated with cystic fibrosis, an autosomal recessive disease, resulting in a live birth. Since then, PGT-M has been used to decrease the chances of propagation of known genetic diseases (Handyside *et al*., 1990; 1992; Zhao *et al*., 2011).

In monogenic diseases, PGT-M is used to detect specific pathogenic variations in the gene sequence associated with certain phenotypes. An example is the association of the ΔF508 mutation and the development of cystic fibrosis (Berger & Baker, 2014). Many genetic variations produce heterogeneous phenotypes in different people due to variable penetrance and expression. However, it is appropriate to offer PGT-M when a parent is known to have a specific DNA variation that may have deleterious effects on the phenotype of their offspring (Brezina & Kutteh, 2015).

Before performing the test, the inheritance pattern of the genetic variation in question must be defined. For example, cystic fibrosis is an autosomal recessive disorder. Therefore, if one spouse has a single mutation ΔF508 and the other does not have any known genetic variation that predisposes their offspring to cystic fibrosis, PGT-M is not indicated. However, if both parents carry a mutation for cystic fibrosis, PGT-M is indicated. In contrast, dominant autosomal disorders usually require testing, even if only one parent has the disease - the case in Huntington's disease. Similarly, women with X-linked recessive disorders should also be counseled about the availability of the test (Berger & Baker, 2014; Janssens *et al*., 2014; Van Rij *et al*., 2012; Verlinsky *et al*., 1992; 2004).

### Monogenic diseases

Genotyping and direct sequencing are the most common methods used to identify monogenic diseases. As only one or a few cells are harvested during biopsy, both techniques require DNA amplification. This has traditionally been done through PCR protocols (Berger & Baker, 2014). More recently, however, some centers have achieved high-quality DNA amplification for monogenic diseases and a screening of 23 pairs of chromosomes using a modified genome- wide amplification protocol (Rechitsky *et al*., 2013).

A recent technology called karyomapping uses broad genomic linkage analysis to compare SNPs of the couple to SNPs of family members with known genetic statuses to identify the combination of SNP alleles associated with a chromosome carrying the genetic mutation. In this method, a monogenic disease can be identified without knowledge of the specific associated genetic mutation. Karyomapping has shown high accuracy and presents 97.7% agreement with conventional PGT-M without the need to design specific tests for any disease (Natesan *et al*., 2014). In addition to the detection of cystic fibrosis and Huntington's disease, Table 4 lists other possible diseases to be analyzed with PGT-M (Sullivan-Pyke & Dokras, 2018).

**Table 4:** Monogenic diseases diagnosed by PGT-M

Dominant Autosomal Diseases	Recessive Autosomal Diseases	X-Linked Diseases
Familial adenomatous polyposis	Sickle-cell anemia	Duchenne muscular dystrophy
Huntington's disease	Spinal muscular atrophy	Becker muscular dystrophy
Breast cancer (BRCA1/BRCA2) mutations	Joubert syndrome	Chronic granulomatous disease
Retinoblastoma	Osteogenesis imperfecta	Fragile X syndrome
Kell antigen system	Gaucher disease	X-linked adrenoleukodystrophy
Myotonic dystrophy	Fanconi syndrome	
Peutz-Jeghers syndrome	Propionic acidemia	
Dilated cardiomyopathy	Cystic fibrosis	
Lynch syndrome	Homocystinuria	
Crouzon syndrome	Usher syndrome	
Polycystic kidney disease	Familial dysautonomia	
Brugada syndrome	Methylmalonic acidemia	
Multiple endocrine neoplasia	Alpha-1 antitrypsin deficiency	
Hereditary multiple osteochondromas		

Source: Sullivan-Pyke & Dokras, 2018.

### Chromosomal alterations

PGT-M can also be used in parents with known structural chromosome aberrations. These aberrations may be present in the form of translocations (reciprocal or Robertsonian) or inversions (mainly pericentric, but also paracentric to a lesser degree) (Escudero *et al*., 2008).

Reciprocal translocations usually involve the breaking and reunion of two different chromosomes with exchange of the acentric terminal segments. Robertsonian translocations involve the fusion of two acrocentric chromosomes and the loss of the short arms of these chromosomes. It is worth mentioning that the short arms of acrocentric chromosomes (pairs 13, 14, 15, 21, and 22) supposedly contain little genetic information of relevance. Chromosome inversions are two breaks in the same chromosome, either in the same arm (paracentric) or one in each arm (pericentric), with inversion of the segment between the points of interruption (Escudero *et al*., 2008; Lim *et al*., 2008).

People with such structural chromosomal aberrations usually have a normal phenotype because all the necessary genetic coding is present, though not organized in the standard way. These aberrations are therefore referred to as translocations or balanced inversions or inversions. However, the descendants of these individuals are at higher risk of unbalanced translocations or inversions (Escudero *et al*., 2008; Lim *et al.,* 2008).

The chances of a child having an unbalanced karyotype depend on the type of structural chromosomal aberration of the parents and, possibly, the gender of the parent carrier. Unbalanced translocations in offspring usually result in pregnancy loss or severe birth defects (Bint *et al*., 2011).

Structural chromosomal aberrations are present in less than 1% of phenotypically normal adults, but are detected in one partner in 2-5% of couples with a history of recurrent pregnancy loss. However, most American specialists and medical societies recommend parental karyotyping as part of the diagnostic investigation of couples with recurrent miscarriages (ASRM, 2012; Brezina & Kutteh, 2014).

Conventional techniques for detecting chromosomal aberrations use FISH, but the method has severe limitations. These include errors resulting hybridization and errors tied to the complexity of the testing procedure, which increase significantly with the test is not performed by a trained specialist. In addition, FISH generally does not assess the ploidy status of chromosomes that are not part of the known structural aberration. In many patients with such aberrations, embryos may be balanced for the chromosome in question, but still harbor aneuploidies in other chromosomes. Therefore, technologies based on SNP and NGS are currently preferred (Brezina & Kutteh, 2014; Harper & Sengupta, 2012; Sullivan-Pyke & Dokras, 2018).

Although carriers of recessive conditions and carriers of balanced chromosome rearrangements do not have genetic disease, their offspring may be at increased risk of being affected. PGT-M can help individuals in this situation to have the same chance of bearing a healthy child as the general population. The technique is an option among other available reproductive options, which also include gamete donation and adoption (Brezina & Kutteh, 2014). The use of PGT-M to prevent the spread of parental disorders of genetic origin is recognized by professionals and international societies as an appropriate medical procedure (Ferraretti *et al*., 2013; Ginsburg *et al*., 2011; Harton *et al*., 2011).

The number of patients eligible for the test is likely to increase in the coming decades, as the number of diseases with an identifiable genetic cause continues to increase. Currently, many of the mutations assessed by PGT-M lead to specific syndromes, such as cystic fibrosis. However, it is now known that many common diseases, such as diabetes mellitus, hypertension, and breast cancer, are associated with certain gene sequences or mutations. In the future, the test might be used to detect genetic sequences or mutations that predispose to certain diseases (Brezina, 2013; Cirulli & Goldstein, 2010; Harper *et al*., 2013).

### The ethics of embryo biopsy in Brazil

Resolution 2168/2017 of the Brazilian Board of Medicine regulates preimplantation genetic testing at a federal level. These technologies cannot be used to select the sex or any other biological characteristic of the future child, except in cases in which this is done to avoid diseases in the offspring. After the embryos have been selected for transfer, the remaining embryos can be discarded or donated for research upon written consent by the couple. Preimplantation genetic testing can also be used to type embryo HLA in order to select embryos that are HLA-compatible with a sibling affected by a disease which treatment is stem cell transplantation (CFM, 2017).

## CONCLUSION

The interface between genetics and human reproduction has become increasingly larger, as knowledge about the genetic causes of infertility grows and the availability of genetic testing in daily clinical practice increases. Genetic tests often enable the identification of the cause of infertility and increase the success rate of fertility treatments. It is also an important tool in counseling individuals at risk of early loss of reproductive capacity and couples with genetic alterations. Genetic testing should also be considered in investigations of recurrent pregnancy loss and as part of gamete donor screening procedures. Preimplantation genetic testing is fundamental to avoid the occurrence of severe diseases in children of couples at increased risk. However, ordering tests and performing treatment must always be based on sound research performed to evaluate efficacy, safety (including long-term), and cost-effectiveness. This continually evolving field requires close communication between clinical genetics, IVF teams, and patients to ensure that everyone is fully informed and able to make well thought out choices.

The success rates of assisted reproductive technology procedures are increasing, and genetic diagnosis is a fundamental element in the treatment of infertile couples. Further discussions are required about which procedures are clinically and ethically acceptable and how they should be regulated.
